# An Update on Omega-3 Polyunsaturated Fatty Acids and Cardiovascular Health

**DOI:** 10.3390/nu13010204

**Published:** 2021-01-12

**Authors:** Andrew Elagizi, Carl J. Lavie, Evan O’Keefe, Keri Marshall, James H. O’Keefe, Richard V. Milani

**Affiliations:** 1 Department of Cardiovascular Diseases, John Ochsner Heart and Vascular Institute, Ochsner Clinical School, The University of Queensland School of Medicine, New Orleans, LA 70121, USA; andrew.elagizi@ochsner.org (A.E.); rmilani@ochsner.org (R.V.M.); 2 Tulane Medical Center, New Orleans, LA 70112, USA; eokeefe3@tulane.edu; 3 Director Medical and Scientific Communications, Pharmavite LLC., West Hills, CA 91304, USA; kmarshall@pharmavite.com; 4 Saint Luke’s of Kansas City, Mid America Heart Institute, University of Missouri, Kansas City, MO 64111, USA; jokeefe@saint-lukes.org

**Keywords:** omega 3 polyunsaturated fatty acid, omega 3 index, cardiovascular disease

## Abstract

Interest in the potential cardiovascular (CV) benefits of omega-3 polyunsaturated fatty acids (Ω-3) began in the 1940s and was amplified by a subsequent landmark trial showing reduced CV disease (CVD) risk following acute myocardial infarction. Since that time, however, much controversy has circulated due to discordant results among several studies and even meta-analyses. Then, in 2018, three more large, randomized trials were released—these too with discordant findings regarding the overall benefits of Ω-3 therapy. Interestingly, the trial that used a higher dose (4 g/day highly purified eicosapentaenoic acid (EPA)) found a remarkable, statistically significant reduction in CVD events. It was proposed that insufficient Ω-3 dosing (<1 g/day EPA and docosahexaenoic acid (DHA)), as well as patients aggressively treated with multiple other effective medical therapies, may explain the conflicting results of Ω-3 therapy in controlled trials. We have thus reviewed the current evidence regarding Ω-3 and CV health, put forth potential reasoning for discrepant results in the literature, highlighted critical concepts such as measuring blood levels of Ω-3 with a dedicated Ω-3 index and addressed current recommendations as suggested by health care professional societies and recent significant scientific data.

## 1. Introduction

As early as 1944, Sinclair described the rarity of coronary heart disease (CHD) amongst Greenland Eskimos, who consumed a diet rich in fish, seal and whale [1]. More than 40 years ago, Bang and Dyerberg reported that despite low consumption of fruit, vegetables and complex carbohydrates in exchange for a diet high in saturated fat and cholesterol, serum cholesterol and triglyceride (TG) levels were lower in Greenland Inuit than in age-matched residents of Denmark, who also demonstrated lower risk of myocardial infarction (MI) [2]. These and other similar observations sparked interest in the potential benefits of increased dietary fish intake, particularly the benefits of omega-3 polyunsaturated fatty acids (Ω-3), for cardiovascular (CV) health.

Ω-3 confer CV benefits through TG reduction, anti-inflammatory and anti-arrhythmic effects, vasodilation, reduced blood pressure, improved arterial and endothelial function, favorable autonomic tone, and reduced platelet aggregation [3,4,5]. In particular, TG levels are a historically well-studied, independent risk factor for CHD. Ω-3 or fish oil diet supplementation is evidenced to lower TG levels in a dose-dependent fashion, whereby 3–4 g/day of eicosapentaenoic acid (EPA) or a combined EPA and docosahexaenoic acid (DHA) reduces blood levels by 20–50% in those with high TGs [6].

## 2. Controversies Surrounding Ω-3

The initial landmark trial for Ω-3 supplementation, GISSI-P, showed that 1 g/day Ω-3 (EPA + DHA) decreased the risk of death, non-fatal acute MI and stroke in patients with recent MI (<3 months) [7]. With approximately 11,000 subjects, almost 4 years of follow up with risk reduction of 14% in the combined primary endpoint and an overall 20% reduction in fatal events, there was initial excitement. However, multiple subsequent studies have failed to corroborate those results.

Since GISSI-P was published, however, there have been several fundamental developments in CV disease (CVD) research; for example, how intervention study arms are treated with aggressive medical therapy. Only 5% of GISSI-P patients received statin therapy at baseline [8], in contrast to the OMEGA trial, in which 81% of patients received statin therapy at baseline. The lack of an Ω-3 benefit in the OMEGA trial may have been due to the low rate of sudden cardiac death, total mortality and major adverse CV events (MACE) within 1 year follow up after MI in those that received this improved optimal medical therapy [9].

Further controversy resulted from articles in popular media which wrongly suggested that Ω-3 ingestion, including that from fish, increased prostate cancer risk, with many commentaries thereafter advising against the use of supplemental fish oil [10]. Habitual high intake of fish and seafood in men who have prostate cancer, however, has been linked to significantly improved survival. Accordingly, Japanese men, who consume approximately 8 times more fish than their American counterparts, have a rate of prostate cancer mortality many-fold lower [10]. Ultimately, a more thorough review of the literature would suggest that increased Ω-3 consumption does not increase prostate cancer risk, but decreases prostate cancer mortality in addition to reducing sudden cardiac death and CV events [11,12].

It is also important to consider the populations who show beneficial findings from Ω-3 intake as well. Trials such as GISSI-P and JELIS were conducted in Italian and Japanese populations [7,13], respectively, who have higher baseline Ω-3 levels due to lifestyles composed of increased fish and seafood intake as compared to the typical diet of Western populations. One possibility is that there may exist a threshold effect regarding endogenous Ω-3 levels and CVD benefits, as some studies have shown that participants with lower baseline Ω-3 levels have more impactful results after supplementation. Therefore, populations such as those in GISSI-P and JELIS who may consume more fish on a regular basis and therefore have higher baseline Ω-3 levels may require a lower dose of Ω-3 to achieve a clinical benefit, as opposed to Western populations who on average consume less fish and likely require a higher Ω-3 dose to achieve the same therapeutic benefit. Supporting this notion are the findings of the REDUCE-IT trial [14], in which higher Ω-3 doses (4 g/day of highly purified EPA) were associated with significant CVD benefits, and, in particular, a more robust benefit in the US cohort.

## 3. Recent Data

In 2018, three large trials evaluating Ω-3 and CV health were released, with divergent conclusions. The ASCEND trial found no reduction in CVD risk when 1 g/day EPA + DHA was used for primary prevention in patients with diabetes [15]. There was no significantly lowered incidence of serious vascular events in 15,480 patients after mean 7.4 years of follow up. Not to be overlooked, however, there was a statistically significant 18% relative risk reduction in vascular death, defined as death from CHD, stroke or other vascular causes, which seems to be a meaningful endpoint that was not emphasized in this paper.

The VITAL trial administered 2000 IU/day vitamin D3 and 1 g/day Ω-3 (EPA + DHA) for primary prevention of CVD and cancer in 25,871 patients and found no difference between the intervention and placebo groups at a median of 5.3 years [16]. Nevertheless, this trial, similar to ASCEND, also found a statistically significant reduction in total MI which carried a hazard ratio (HR) of 0.71; 95% confidence interval (CI) 0.59–0.9 [16]. Patients consuming fewer than 1.5 fish meals per week, who then received Ω-3 supplementation, had a significant reduction in MACE by 19% and risk of MI by 40%, suggesting the importance of baseline and/or threshold Ω-3 levels to achieve a therapeutic benefit.

The REDUCE-IT trial administered icosapent ethyl (highly purified EPA formulation) at 4 g/day to 8179 patients with established CVD or diabetes receiving statin therapy with TG levels of 135–499 mg/dL and low-density lipoprotein levels of 41–100 mg/dL [14]. The primary endpoint (composite of CVD death, non-fatal MI, non-fatal stroke, CV revascularization or unstable angina) was reduced by 25% in the treatment group with a number needed to treat (NNT) of 21 and reduced the secondary endpoint of MACE by 26% with a NNT of 28. The subgroup including patients in the US demonstrated a HR of 0.74; 95% CI, 0.66–0.83 for the primary efficacy composite endpoint. The cohort of patients from the US demonstrated a statistically significant 30% relative risk reduction and 2.6% absolute risk reduction in all-cause mortality (NNT of 39) [17]. While the non-US subgroup also showed significant reductions in the primary and key secondary composite endpoints, the US subgroup demonstrated particularly robust risk reductions. However, the treatment group did experience higher hospitalizations for atrial fibrillation or flutter and serious bleeding.

**Figure 1:**
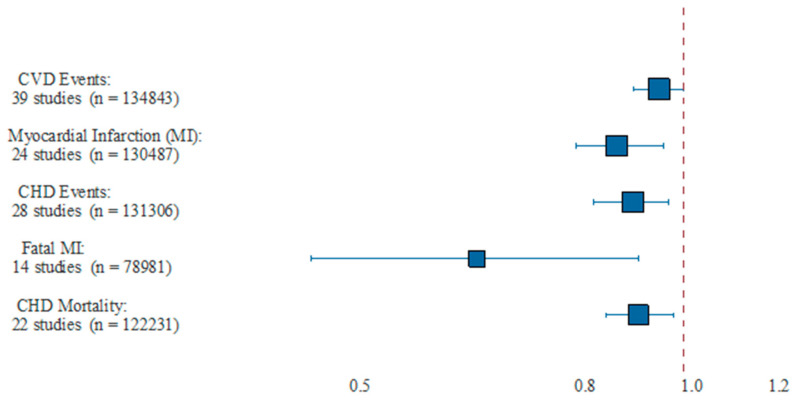
Pooled results from meta-analysis. This figure showed the pooled estimate of relative risk and 95% CI, as well as the number of studies and combined number of participants. CHD = coronary heart disease; CVD = cardiovascular disease; MI = myocardial infarction. Reproduced with permission from: Bernasconi, A.A., Wiest, M.M., Lavie, C.J., Milani, R.V., Laukkanen, J.A. Effect of Omega-3 Dosage on Cardiovascular Outcomes: An Updated Meta-Analysis and Meta-Regression of Interventional Trials. *Mayo. Clin. Proc*. 2020; S0025-6196(20)20985-X.

**Figure 2:**
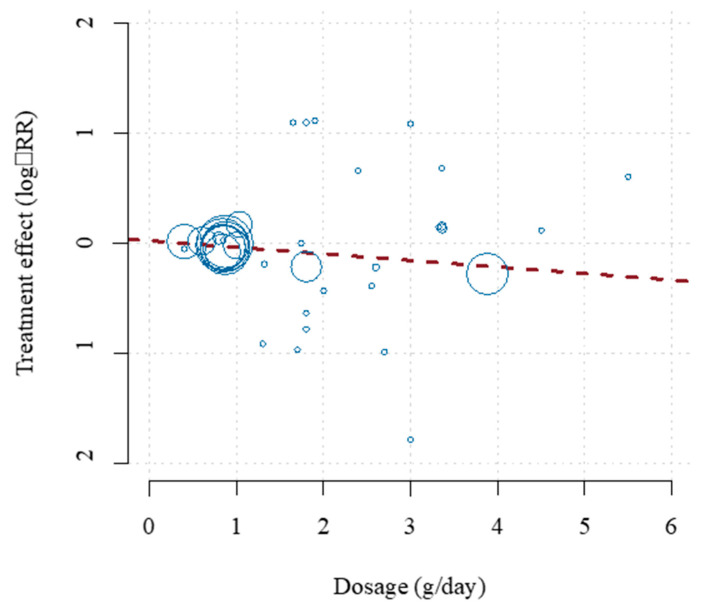
Dose–effect relationship for the prevention of CVD events. The horizontal axis shows the EPA + DHA dosage in grams per day. The vertical axis shows the treatment effect (log-relative risk). The area of each study square is proportional to its regression weight (inverse-variance of relative risk estimate). Reproduced with permission from: Bernasconi, A.A., Wiest, M.M., Lavie, C.J., Milani, R.V., Laukkanen, J.A. Effect of Omega-3 Dosage on Cardiovascular Outcomes: An Updated Meta-Analysis and Meta-Regression of Interventional Trials. *Mayo. Clin. Proc*. 2020; S0025-6196(20)20985-X.

Three recent meta-analyses demonstrated equally inconsistent results [18,19,20]. Unlike previous studies, a meta-analysis by Bernasconi et al. [21] used all available evidence in measuring the effect of Ω-3 dosage and was the first to use meta-regression to examine other potential sources of heterogeneity in prior studies. EPA + DHA was associated with statistically significant lowered risk of CHD events and MI with equivalent risk reductions of 9% and 13%, respectively. Importantly, this risk reduction was dose dependent for MI, as each additional 1 g/day was associated with a significant risk reduction of 9%. The benefits of a 35% reduction in fatal MI and 9% reduction in CHD mortality tended to occur at lower doses (<800 to 1200 mg/day), which agrees with the findings of Mozaffarian and Rimm [22]. Ultimately, this study found reduced risk of MI (NNT of 272), CHD events (NNT of 192), fatal MI (NNT of 128) and CHD mortality (NNT of 431). The pooled analysis of CV events and dose–response effect from this meta-analysis are shown in Figure 1 and Figure 2, respectively. This analysis revealed that EPA + DHA supplementation results in a statistically significant risk reduction for the outcomes of MI, CHD events, fatal MI and CHD mortality. For CVD events and MI, the slope is negative and significantly non-zero (*p* < 0.01), indicating that the higher dosages used in the included studies are associated with increased protection [21]. For CVD events, the estimates for the slope translate to a risk reduction of 5.8% for each additional 1 g/day intake, and 9% risk reduction for MI. This paper also did not find any statistical benefit of total EPA dosage compared with combined EPA/DHA dosage, nor did it find a significant association between year of publication and overall impact of EPA/DHA to improve CVD outcomes, meaning the more recent studies showed similar overall benefits as did the older studies when optimal medical therapy was less advanced (Figure 1 and Figure 2).

Another recent meta-analysis of 13 trials demonstrated that Ω-3 supplementation lowers the risk of MI, CHD death, total CHD, CVD death and total CVD, after excluding REDUCE-IT from the analysis [23]. Including REDUCE-IT in the analysis resulted in an even stronger inverse association for these outcomes, though REDUCE-IT introduced significant heterogeneity (due to the substantially higher treatment dose of 4 g/day in this trial).

## 4. The Critical Importance of Dosage

There is mounting evidence that higher doses of Ω-3 interventions appear more likely to demonstrate CVD and other clinical benefits. Deficiencies of many nutrients can lead to debilitating and life-threatening chronic disease, which can be corrected with repletion of the deficient nutrient. Supplementation, on the other hand, does not have as profound an effect in people who are nutrient replete. For example, administration of thiamin in wet Beri-Beri can be curative and life-saving [24], whereas supplementation in patients with normal thiamin levels would not be so dramatic. Likewise, studies such as VITAL have shown the most CVD benefit in patients with the lowest baseline levels of Ω-3 [16], who may represent the ideal population for this therapy.

High-dose Ω-3 appeared to be beneficial in multiple studies, in addition to the results produced by REDUCE-IT. OMEGA-REMODEL found that 4 g/day Ω-3 (EPA + DHA) for 6 months after acute MI demonstrated a reduction in adverse left ventricular remodeling, non-infarct myocardial fibrosis and serum biomarkers of systemic inflammation beyond the current guideline-based standard of care [25]. Further, the finding that Ω-3 EPA + DHA levels >1.5 g/day are typically required for a clinically meaningful change in TG levels further explains the inconsistent results in trials using lower doses (e.g., 1 g/day) [18]. Goodfellow et al. [26]. Administered Ω-3 of EPA + DHA at 4 g/day and measured endothelial function at baseline and after 4 months of therapy, finding a significant improvement in arterial flow-mediated dilation along with significant TG reduction whereas placebo led to no change in either parameter.

Heart transplant patients who received 4 g/day Ω-3 versus placebo as prophylaxis against cyclosporine-induced hypertension demonstrated a systolic blood pressure reduction of 2 +/− 4 mm Hg in the Ω-3 group versus an increase in 17 +/− 4 mm Hg in the placebo group, with a diastolic increase of 10 +/− 3 mm Hg and 21 +/− 2 mm Hg in the treatment and placebo groups, respectively, following 6 months of treatment [27]. The authors concluded that Ω-3 supplementation at 4 g/day (EPA + DHA) was effective for prophylaxis against cyclosporine induced hypertension, which occurs in a high proportion of patients following heart transplantation [28].

**Figure 3:**
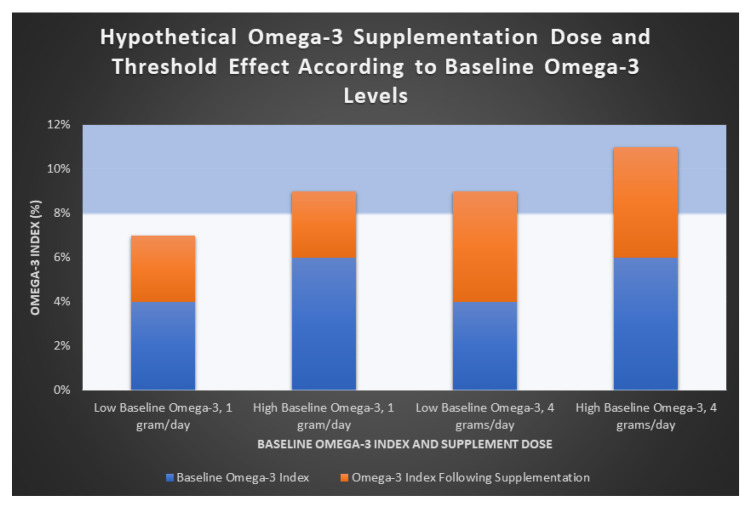
Hypothetical omega-3 supplementation dose and threshold effect.

In summary, the populations of Japan and Italy in the JELIS and GISSI-P trials, respectively, who demonstrated significant CVD benefits with lower-dose (1–2 g/day) Ω-3 EPA + DHA may have demonstrated such due to a “threshold level” which reaches the therapeutic window of Ω-3. This can explain why higher doses (e.g., 4 g/day) may be required to demonstrate CV benefit, particularly in studies of American populations with lower baseline Ω-3 levels. Figure 3 demonstrates a hypothetical Ω-3 threshold effect with supplement doses of 1 g/day and 4 g/day of EPA + DHA. While GISSI-P originally showed CVD protective effects with an Ω-3 formulation of EPA + DHA, REDUCE-IT and JELIS showed quite dramatic effects using EPA alone. However, a recent large meta-analysis found no statistical difference between the total EPA or combined EPA + DHA dosage on the effects on MACE [21].

This figure demonstrates a hypothetical representation of the proposed threshold effect of Ω-3. An Ω-3 index > 8% has been suggested to predict lower CV risk [6]. Groups are shown according to both low and high baseline Ω-3 levels (blue bars). Increases in Ω-3 index achieved with both low-dose (1 g/day) and high-dose (4 g/day) supplementation of Ω-3 EPA + DHA are shown (orange bars). In the low supplementation group (1 g/day, first two columns), only patients with high baseline Ω-3 levels reached the 8% therapeutic threshold, suggesting that a dose of 1 g/day of EPA + DHA may be ineffective in those with lower baseline Ω-3 levels. In those receiving a higher dose (4 g/day of EPA + DHA, columns 3 and 4), both groups are more likely to reach the therapeutic threshold. This is a hypothetical representation and further study is required to support this theory.

## 5. Ω-3 Index

One aspect that has been lacking from this field of research for a long time, however, is a concerted methodology of measurement. Hereto now, most research has utilized average fish consumption or arbitrary supplement dosing, often not considering this source of Ω-3 or the individual’s initial or concluding Ω-3 blood levels. The Ω-3 index is a measurement of serum Ω-3 levels (EPA + DHA), with multiple potential uses in research and clinical practice. The Ω-3 index has been proposed as an indicator of increased CHD risk when <4% [6], which also coincidently reflects the estimated average American serum level of Ω-3 [29]. An individual is at low risk when their Ω-3 index is >8% [30,31]; and studies have shown that achieving an Ω-3 index > 8% can potentially reduce the risk of fatal CHD by approximately 35% [29,32]. A large meta-analysis of global studies using biomarkers of Ω-3 in 45,637 patients without CHD revealed that higher Ω-3 levels are strongly correlated with lower incidence of fatal CHD [33]; this is an inverse relationship whereby higher Ω-3 levels are associated with lower risk of CHD in a continuous gradient fashion. Just as hemoglobin A1c is the clinical standard for assessing glycemic status, the Ω-3 index is a superior method for evaluating long-term Ω-3 status [29]. Adequate diagnostics for indicating Ω-3 intake is critical to ensuring a personalized approach to prescribing EPA and DHA for an individual to achieve health outcomes. For clear reasons, this index can also be used as a target in clinical trials to reduce heterogeneity in trial design. Effective use of the Ω-3 index as a clinical diagnostic and research tool may be the key to resolving the controversy surrounding Ω-3 therapy.

## 6. Heart Failure

The benefits of Ω-3 have also been studied in heart failure (HF) patients, and the most impressive study focusing on Ω-3 in this population was the GISSI-HF trial [32]. This study, conducted in Italy, involved 1 g/day Ω-3 (EPA + DHA) or placebo in nearly 7000 HF patients (91% with reduced ejection fraction (HFrEF)) followed for a median of 3.9 years. Not only did this trial confirm the safety of Ω-3 in HF patients, but also found a NNT of 56 over 3.9 years to prevent one death or NNT of 44 to avoid one death or hospital admission for CVD reasons. Based on the results of GISSI-HF, the American Heart Association (AHA) provides a class IIa indication that Ω-3 treatment is reasonable among patients with HFrEF [33].

However, the AHA does not provide recommendations for Ω-3 use in the primary prevention of HF due to a lack of data [33]. A meta-analysis of 7 prospective studies with 176,441 subjects and 5480 incident cases of HF found a lower risk of HF with higher intake of marine Ω-3 (Relative risk 0.85; 95% CI, 0.73–0.99, *p* = 0.04) [34]. Block et al. [35]. measured EPA levels in 6,562 patients over 13 years, finding that plasma EPA levels were significantly lower in HF patients compared to HF-free patients (*p* = 0.005), and this benefit was noted among patient groups with either preserved or reduced ejection fractions. The possibility that increased Ω-3 may reduce HF incidence warrants further study. An AHA scientific advisory suggests a general dietary recommendation for 1–2 fish servings per week to prevent HF [36].

Not only is it possible that Ω-3 can reduce HF incidence, Ω-3 may be beneficial in end-stage HF as well. A small study of 14 patients with New York Heart Association class III–IV HF received 8 g of Ω-3 vs. placebo for 18 weeks, showing a significant 59% reduction in tumor necrosis factor-α (TNF-α) levels, and 39% decrease in interleukin-1 (IL-1), and body weight increase (due to decreased cachexia), whereas the placebo group demonstrated a significant 44% increase in TNF-α and no change in IL-1 [37]. While underpowered, these findings show promise that Ω-3 may represent a novel therapeutic approach in treating end-stage HF with cachexia. These mechanisms of decreased inflammation may explain the beneficial findings of GISSI-HF.

Effects of Ω-3 have also been studied extensively in various forms of CVD, with several studies demonstrating improvements in CVD risk factors, including atherosclerosis, atrial and ventricular arrhythmias, hyperlipidemia, peripheral arterial disease and ischemic stroke [3]. An extensive discussion regarding the benefits of Ω-3 therapy for these conditions is beyond the scope of this article and has been discussed previously [3,4,33]. Studies regarding Ω-3 and HF have been emphasized here due to recent HF guidelines suggesting that Ω-3 treatment is reasonable among HFrEF patients, and mounting evidence that suggests Ω-3 may be beneficial in reducing HF incidence as well.

## 7. Molecular Mechanisms

The mechanisms responsible for the CVD benefits of Ω-3 intake have not been clearly established, though recent research provides new insights. Some CVD Benefits from Ω-3 therapy may be attributable to metabolites which are potent anti-inflammatory mediators. For example, the resolvin E series of metabolites are synthesized from EPA and actively reduce leukocyte tracking to the site of inflammation, promote the clearance of inflammatory cells and suppress cytokine production [38]. Multiple animal studies have shown the benefits of these anti-inflammatory mediators post-MI [39]. Administration of resolvin E1 attenuates the infarct size in rats subject to ischemia/reperfusion injury and resolvin D1 induces a switch to anti-inflammatory M2 macrophages in the left ventricle to prevent myocardial fibrosis [40,41].

In addition to anti-inflammatory effects, Ω-3 may reduce arrhythmias via direct inhibition of sarcolemmal ion channels which may stabilize electrical activity and prolong the relative refractory period of the cardiomyocytes [42]. Anti-arrhythmic effects have been demonstrated in both animal as well as human studies. EPA dose dependently reduced pulmonary vein spontaneous beating and the amplitude of delayed afterdepolarizations in rabbit tissue [43]. Prolonged atrial refractoriness has been shown in humans with Ω-3 supplementation (6 g/day EPA + DHA for at least 1 month) with reduced vulnerability to induce atrial fibrillation [44], though trials such as STRENGTH and REDUCE-IT showed increased rates of atrial fibrillation. It is possible that higher doses, such as 6 g/day, may be needed for some anti-arrhythmic effects. Ω-3 also improve endothelial function by increasing nitric oxide production by directly stimulating endothelial nitric oxide synthase gene and protein expression [45]. Ongoing research regarding these molecular pathways is required to better understand the CVD benefits of Ω-3.

## 8. Dietary Sources and Guidelines

Fish oil is obtained in the human diet through seafood, specifically oily fish, such as salmon, sardines, herring, mackerel, albacore tuna, and trout [3,29], or Ω-3 supplementation. Ω-3 levels are naturally high in wild fish, whereas farm-raised fish tend to be grain fed and have resultant lower Ω-3 levels. Ω-3 are not synthesized de novo, rather they stem from unicellular organisms such as algae, at the base of the marine food chain [12]. Fish consume algae in the wild and as a result compose their own Ω-3, whereas most farm-raised fish (including tilapia and catfish) are not fed this natural diet and thus have low Ω-3 levels. However, farmed Atlantic salmon, because they are fed ground fish meal, have Ω-3 as high or higher than wild salmon. On the other hand, fish that are naturally low in fat, such as cod, scallops, lobster, mussels and shrimp, contain lower levels of Ω-3 [29]. Generally, shark, swordfish, tilefish and king mackerel contain high levels of mercury and frequent ingestion should be avoided [29]. Of note, mercury is water soluble and protein bound, and is therefore present in the muscle of fish but not as readily in the oil; as a result of this and high-quality manufacturing, fish oil supplements contain negligible amounts of mercury [3].

Plant sources of Ω-3, primarily in the form of alpha-linolenic acid, include soybean, flaxseed, chia, hemp, linseed, rapeseed (canola oils) and tree nuts [5]. Because plant Ω-3 are converted to EPA and DHA in limited amounts, plant-based Ω-3 should not be considered a replacement for preformed Ω-3 EPA and DHA. Further study is required to better understand the role of plant-based Ω-3, and how they may play a stronger role in health and may also be a sustainable source, such as in the case of algae, which does naturally contain EPA and DHA [4].

The current (2015–2020) dietary guidelines for Americans recommend consumption of approximately 8 ounces per week of a variety of seafood for the general population, which would be expected to provide an average daily intake of approximately 250 mg of EPA and DHA [46]. That amount of 250 mg/day is associated with reduced cardiac deaths among individuals with and without pre-existing CVD [46]. The 8 ounces of seafood or 2 servings per week can be sustainable and environmentally friendly and is recommended by the AHA [36]. However, that amount will not increase the Ω-3 index to a level > 8%. A daily intake of between 1 and 2 g/day of Ω-3 EPA + DHA is required to achieve an Ω-3 index > 8%, and an individual’s ultimate dose is largely dependent on their background diet of other fats, specifically omega-6 fatty acids.

The 2019 American College of Cardiology/AHA guidelines for the primary prevention of CVD offer a class I recommendation for a diet emphasizing the intake of vegetables, fruits, legumes, nuts, whole grains and fish to decrease CVD risk factors [47]. A 2018 science advisory from the AHA regarding Ω-3 recommends consumption of non-fried seafood 1–2 times per week for CVD benefits, including reduced risk of CVD death, CHD, and ischemic stroke [36]. Recently, a Pesco-Mediterranean diet (a traditional Mediterranean diet, with fish as the primary animal protein source) with time-restricted food intake (intermittent fasting) has been proposed to be beneficial for CV and neurologic health, though prospective and randomized studies are needed to confirm efficacy [48].

## 9. Late-Breaking Trials

Two Ω-3 trials were released in late 2020, which did not find any CV benefits of Ω-3 therapy in high-risk patients. OMEMI used a lower-dose intervention (1.8 g/day EPA + DHA in addition to standard medical care) in elderly patients (age 70–82) following acute MI (2–8 weeks) [49]. There was no statistically significant difference in outcomes between treatment and placebo groups, which may be explained by insufficient Ω-3 dosing.

The results of the STRENGTH trial add substantially more confusion. Participants in this international study had dyslipidemia, high TG and low levels of high-density lipoprotein cholesterol, and received 4 g/day of a carboxylic acid formulation of EPA + DHA or corn oil placebo in addition to standard medical therapy [50]. The study was prematurely ended after 1384 patients experienced a primary endpoint (composite CV death, non-fatal MI, non-fatal stroke, coronary revascularization or unstable angina) due to a low probability of clinical benefit using this carboxylic acid Ω-3 formulation, according to an interim analysis. There was no significant difference in the primary endpoint occurrence between groups.

The stark difference in findings between STRENGTH (no benefit of 4 g/day carboxylic acid formulation of EPA + DHA) and REDUCE-IT (robust CV benefit using 4 g/day highly purified EPA) add to the confusion surrounding the topic of Ω-3 and CV health. It has been argued that the placebo group receiving mineral oil in the REDUCE-IT trial may have had increased harm caused by the mineral oil, which was associated with >30% increase in C-reactive protein levels [50]. However, the FDA subsequently awarded a label claim for CVD event reduction for icosapent ethyl based on analyses that concluded that the effects of mineral oil could not entirely explain the observed differences in outcomes. It is also possible that the DHA component of the STRENGTH intervention may have led to harm in the treatment group. However, this is unlikely as DHA has not demonstrated adverse CVD events and DHA concentrations were modest and did not correlate with event rates. STRENGTH and REDUCE-IT both reported increased atrial fibrillation events. Our recent meta-analysis is being updated to include these studies, and although the overall results are slightly weakened, the overall findings remain strongly in favor of Ω-3 improving CVD outcomes [21].

## 10. Conclusions

The Ω-3 index is an objective measurement of endogenous Ω-3 levels, specifically for EPA and DHA, and can be used to evaluate: baseline Ω-3 status, response to Ω-3 therapy, as a clinical target for CV health and, if used consistently in clinical trials, can make future study more easily interpretable and comparable. Effective implementation of diagnostics for Ω-3, including use of the Ω-3 index as a clinical and research tool, may be the key to resolving much controversy surrounding the efficacy of Ω-3 therapy.

Multiple trials continue to use an Ω-3 intervention dose of 1 g/day of EPA + DHA, which demonstrated significant CVD benefits in the landmark GISSI-P trial. However, trials demonstrating a benefit with this low dose, such as GISSI-P, GISSI-HF and JELIS, were performed in Italian and Japanese populations with higher baseline Ω-3 intake in their regular diet, which may account for their ability to reach a therapeutic level of Ω-3 which confers CVD benefits. The efficacy of modern medical therapy for CVD can further confound the benefits of Ω-3 supplementation due to reduced overall CVD events. Patients in Western nations or nations with lower Ω-3 intake in general may require higher-dose interventions (e.g., 2–4 g/day of EPA + DHA) to reach a therapeutic effect of Ω-3.

Several decades and countless dollars have been spent studying the relationship between Ω-3 and CVD without reaching a consensus among clinicians. There is, however, clear evidence from multiple studies that higher doses of Ω-3 (2–4 g/day of EPA + DHA) appear to be safe and to reduce CVD events in multiple CVD populations, which warrants further study to conclusively determine the potential benefits of this safe, inexpensive, and well-tolerated therapy.

## Data Availability

Data sharing not applicable.

## Abbreviations

Ω-3	Omega-3 Polyunsaturated Fatty Acids
AHA	American Heart Association
CHD	Coronary Heart Disease
CI	Confidence Interval
CV	Cardiovascular
CVD	Cardiovascular Disease
DHA	Docosahexaenoic Acid
EPA	Eicosapentaenoic Acid
HF	Heart Failure
HFrEF	Heart Failure with Reduced Ejection Fraction
HR	Hazard Ratio
IL-1	Interleukin-1
MACE	Major Adverse Cardiovascular Events
MI	Myocardial Infarction
NNT	Number Needed to Treat
TG	Triglyceride
TNF-α	Tumor Necrosis Factor-α
